# Endocytic Adaptors in Cardiovascular Disease

**DOI:** 10.3389/fcell.2020.624159

**Published:** 2020-12-11

**Authors:** Kui Cui, Yunzhou Dong, Beibei Wang, Douglas B. Cowan, Siu-Lung Chan, John Shyy, Hong Chen

**Affiliations:** ^1^Vascular Biology Program, Boston Children’s Hospital, Boston, MA, United States; ^2^Department of Surgery, Harvard Medical School, Boston, MA, United States; ^3^Department of Cardiology, Boston Children’s Hospital, Boston, MA, United States; ^4^Division of Cardiology, Department of Medicine, University of California, San Diego, San Diego, CA, United States

**Keywords:** epsin, disabled-homolog 2, endocytic adaptor proteins, atherosclerosis, diabetes, inflammation, receptor-mediated endocytosis, clathrin

## Abstract

Endocytosis is the process of actively transporting materials into a cell by membrane engulfment. Traditionally, endocytosis was divided into three forms: phagocytosis (cell eating), pinocytosis (cell drinking), and the more selective receptor-mediated endocytosis (clathrin-mediated endocytosis); however, other important endocytic pathways (e.g., caveolin-dependent endocytosis) contribute to the uptake of extracellular substances. In each, the plasma membrane changes shape to allow the ingestion and internalization of materials, resulting in the formation of an intracellular vesicle. While receptor-mediated endocytosis remains the best understood pathway, mammalian cells utilize each form of endocytosis to respond to their environment. Receptor-mediated endocytosis permits the internalization of cell surface receptors and their ligands through a complex membrane invagination process that is facilitated by clathrin and adaptor proteins. Internalized vesicles containing these receptor-ligand cargoes fuse with early endosomes, which can then be recycled back to the plasma membrane, delivered to other cellular compartments, or destined for degradation by fusing with lysosomes. These intracellular fates are largely determined by the interaction of specific cargoes with adaptor proteins, such as the epsins, disabled-homolog 2 (Dab2), the stonin proteins, epidermal growth factor receptor substrate 15, and adaptor protein 2 (AP-2). In this review, we focus on the role of epsins and Dab2 in controlling these sorting processes in the context of cardiovascular disease. In particular, we will focus on the function of epsins and Dab2 in inflammation, cholesterol metabolism, and their fundamental contribution to atherogenicity.

Endocytosis is the method that cells utilize to uptake material from outside of the membrane to inside of the cells. There are three major forms of endocytosis, phagocytosis, pinocytosis, and clathrin-mediated endocytosis, each involving its own specific cell machinery.

## Clathrin-Mediated Endocytosis

**FIGURE 1:**
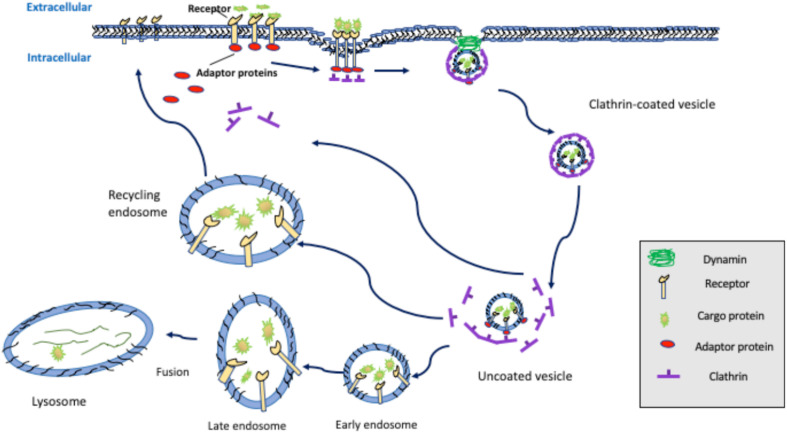
Clathrin-mediated endocytosis. Extracellular ligands binding to cell surface receptors triggers the recruitment of the adaptor proteins, such as AP-2, Dab2, and/or members of the epsin family, which induces plasma membrane invagination and formation of clathrin-coated pits that are subsequently released via dynamin-mediated scission. Clathrin-coated vesicles undergo de-coating and sorting through fusion with early endosomes, late endosomes, and ultimately, lysosomes, leading to receptor degradation, or sorting back to the cell surface by recycling endosomes.

Receptor-mediated endocytosis (i.e., clathrin-mediated endocytosis) is a process by which cells internalize metabolites, hormones, and proteins to allow them to respond to their local environment. This form of endocytosis typically consists of the following steps: (1) extracellular ligand binding to cell surface receptors, (2) the formation of a clathrin cage around the receptor-ligand complex resulting from the interaction with a multitude of molecules and proteins, such as phosphatidylinositol 4,5-bisphosphate (PIP_2_), adaptor protein 2 (AP-2), clathrin-coat assembly protein 180 (AP180), and epsin proteins, (3) lipid bilayer invagination with the aid of membrane curvature promoting proteins, such as members of the epsin family, (4) vesicle formation and release from the plasma membrane, and (5) sorting of the vesicle and receptor-ligand cargo within the cell (Figure 1). Each of these steps requires a variety of endocytic adaptor proteins that include the epsins, epidermal growth factor receptor substrate 15 (Eps15), disabled homolog 2 (Dab2), AP-2, and PIP_2_ (Eberhard et al., 1990; Chen et al., 1998; Pearse et al., 2000; Ford et al., 2002; Polo et al., 2002; Maurer and Cooper, 2005; Bhattacharjee et al., 2020). In this review we focus on the epsin and Dab2 proteins, which play crucial roles in clathrin-mediated endocytosis and are implicated as important modulators of cardiovascular diseases.

## Phagocytosis

Phagocytosis, also called “cell eating,” is used by the cells, such as neutrophils, macrophages and other white blood cells, to engulf debris, bacteria, or other solid objects through the produced pseudopodia (Jaggi et al., 2020; Joffe et al., 2020; Reine et al., 2020; Smirnov et al., 2020). The invagination of cell membrane produces phagosomes, which later fuse with lysosomes containing enzymes. Materials in the phagosome are broken down into simpler substance by these enzymes and degraded.

## Pinocytosis

Pinocytosis is called “cell drinking” that cells, such as hepatocytes, kidney cells and epithelial cells, engulf extracellular fluid, including various solutes, such as sugars, ions, amino acids, and proteins. The cell membrane folds inward to form invagination, which takes up the extracellular fluid and releases it inside the cells. Kidney cell can utilize pinocytosis to separate nutrients and fluid from the urine (Bode et al., 1975). Capillary epithelial cells can use pinocytosis to engulf the liquid part of blood at its surface (Orlov, 1988).

## Epsins

Epsins are a family of adaptor proteins associated with clathrin-coated pits that support lipid bilayer curvature and coordinate the recruitment of ubiquitinated cargo proteins (Ko et al., 2010). The first member of this family to be isolated was epsin 1, which was identified through its interaction with the clathrin-associated protein Eps15 (Chen et al., 1998). Subsequent investigations established that there are three classic members of this family (epsins 1, 2, and 3) in addition to a non-classic isoform named either epsin 4 or epsin R, which is now more commonly referred to as clathrin interactor 1 (CLINT1). In mammals, epsins 1 and 2 are ubiquitously expressed and particularly enriched in the brain (Rosenthal et al., 1999). While epsins 1 and 2 are functionally redundant, epsin 3 is predominantly expressed in the gastric parietal cells of the stomach (Spradling et al., 2001).

**FIGURE 2:**
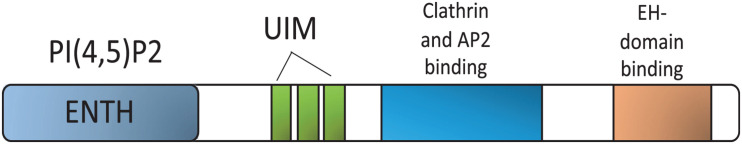
Epsin domain structure. Epsin interacting regions for binding partners. PIP_2_, phosphatidylinositol-4,5-bisphosphate; ENTH, epsin N-terminal homology; UIM, Ubiquitin-interacting motifs; AP-2, Adaptor protein 2; EH, Eps 15 homology domain.

Epsins 1, 2, and 3 share a structure termed the Epsin N-terminal Homology (ENTH) domain, which interacts with PIP_2_ at the plasma membrane (Chen et al., 1999; De Camilli et al., 2002; Chen and De Camilli, 2005). Ubiquitin-interacting motifs (UIMs) that recognize and recruit ubiquitinated surface receptors to clathrin-coated pits for internalization are situated next to the ENTH domain (Oldham et al., 2002; Sakamoto et al., 2004; Chen and De Camilli, 2005). An adjacent region, characterized by DPW (Asp-Pro-Trp)-rich amino acid motifs flanked by a clathrin-binding domain, is responsible for binding to AP-2 and clathrin, respectively (Figure 2). In the COOH-terminal region, NPF (Asn-Pro-Phe) motifs function to bind Eps15-homology (EH) domain-containing proteins, such as Eps15 and the BTB/POZ domain-containing protein POB1 (Salcini et al., 1999; De Camilli et al., 2002).

## Disabled Homolog 2 (Dab2)

Dab2 is another clathrin- and cargo-binding endocytic adaptor protein that was first isolated as a mitogen-responsive phosphoprotein called p96 (Xu et al., 1995). Although the messenger RNA transcript encoding this protein was initially named *DOC2* (for Differentially expressed in Ovarian Cancer) because it was differentially expressed in ovarian cancer, the protein is now referred to as Disabled homolog-2 or Dab2 as it is transcribed from an ortholog of the *Drosophila Dab* gene (Gertler et al., 1989). At the same time, a neuronal-specific isoform named Dab1 represents an additional protein produced from a mammalian ortholog of this *Drosophila* gene (Howell et al., 1997). Interestingly, several spliced isoforms of Dab2 have been identified, including p96 and p67 (Xu et al., 1995; Sheng et al., 2000, 2001) and mammalian Dab2 is expressed in a wide variety of cells and tissues including macrophages, kidney, white adipose tissues, the placenta, and the adrenal gland (Xu et al., 1995; Fazili et al., 1999; Moore R. et al., 2013; Hocevar, 2019). Aside from Dab1, Dab2 shares sequence similarity to other endocytic adaptors, such as Numb, Numbl, and Arh.

**FIGURE 3:**
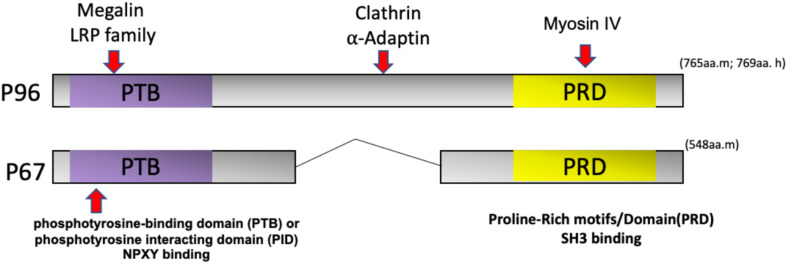
Dab2 domain structure and isoforms. Dab2 has two splice variants called p96 and p67. The NH2-terminal phosphotyrosine-binding domain (PTB) or phosphotyrosine-interacting domain (PID) interacts with receptors, such as the LDL receptor or LDL receptor-related protein (LRP) via the NPXY (Asn-Pro-x-Tyr) motif. The middle region of p96 interacts with endocytic proteins clathrin and α-Adaptin. The COOH-terminal of Dab2 contains a Proline-Rich Domain (PRD) that can bind to Src homology 3 (SH3)-containing proteins. The Dab2 COOH-terminus can also bind myosin VI to mediate endocytic trafficking.

Dab2, Numb, Numblike, and Arh are similar in structure and share an N-terminal phosphotyrosine-binding domain (PTB) or phosphotyrosine-interacting domain (PID) for cargo recognition (Bork and Margolis, 1995; Howell et al., 1999). The PID/PTB domain of Dab2 can interact with transmembrane proteins, such as the LDL and EGF receptors as well as integrins through a NPXY (Asn-Pro-x-Tyr) motif (Morris and Cooper, 2001; Tao et al., 2016a). The interaction between Dab2 and the endocytic proteins clathrin and α-adaptin is mediated by the middle and C-terminal portions of this motif (Traub, 2003). The C-terminus also binds to the motor protein myosin IV (Inoue et al., 2002; Morris et al., 2002a), which facilitates its role in clathrin-mediated endocytosis and trafficking (Figure 3).

## Epsin-Deficient Animal Models

Epsins 1 and 2 are expressed in most vertebrates, contribute to clathrin-mediated endocytosis, and are located near the plasma membrane. Given their ubiquitous expression, their function is strictly cell-type and tissue dependent. Epsins 1 and 2 are widely expressed (Rosenthal et al., 1999), whereas epsin 3 is predominantly expressed in the gastric parietal cells of the stomach (Spradling et al., 2001). As discussed above, epsins are multi-functional proteins that act as endocytic adapters and sort ubiquitinated cargoes. Several studies show that epsins sort ubiquitinated proteins, such as vascular endothelial growth factor receptor-2 (VEGFR2) (Pasula et al., 2012; Dong et al., 2015, 2017), the linear ubiquitin chain assembly complex (LUBAC) (Song et al., 2020), the receptor tyrosine-protein kinase ErbB3 (Szymanska et al., 2016), Notch ligands (Tian et al., 2004; Wang and Struhl, 2004; Chen et al., 2009; Xie et al., 2012; Langridge and Struhl, 2017), and low-density lipoprotein receptor-related protein 1 (LRP1) (Brophy et al., 2019), in addition to playing a role in establishing cell polarity (Li L. et al., 2016). Because epsins are involved in the Notch signaling pathway, which is essential for normal embryonic development, the deficiency of both epsins 1 and 2 results in embryonic lethality. As a result, the study of these proteins in adulthood has relied on inducible knock-out models (Chen and De Camilli, 2005; Chen et al., 2009).

Inducible deficiency of epsins 1 and 2 in endothelial cells in a tumor angiogenesis mouse model revealed the regulatory role of these proteins in vascular development (Song et al., 2017; Dong et al., 2018). More recent studies showed that epsins are involved in regulating Notch signaling to modulate murine embryonic stem cells exit from pluripotency (Cardano et al., 2019). In addition, epsin-mediated Notch signaling has also been reported in another study where the lethality of epsins 1 and 2 double knockout mice die at embryonic day 9.5–10 (E9.5-10), owing to defects in organogenesis, which included failure of blood vessel and heart tube formation and insufficient yolk sac circulation (Chen et al., 2009). Using cell-type and tissue-specific epsin mutant mouse models, the regulatory role of these proteins in development (Chen et al., 2009), tumor angiogenesis (Pasula et al., 2012; Dong et al., 2015), developmental and physiological angiogenesis (Tessneer et al., 2014; Rahman et al., 2016), lymph angiogenesis (Liu et al., 2014; Wu et al., 2018), atherosclerosis (Brophy et al., 2019; Dong et al., 2020), diabetes (Wu et al., 2018), and cancer progression (Pasula et al., 2012; Tessneer et al., 2013a,b; Chang et al., 2015; Dong et al., 2015; Song et al., 2017) have become better understood.

## Epsins in Cardiovascular Disease

Atherosclerosis, a leading cause of morbidity and mortality in cardiovascular diseases, is a multi-factorial and chronic inflammatory disease (Lusis, 2000; Falk, 2006; Yin et al., 2013; Fang et al., 2014). It is characterized by low-density lipoprotein (LDL) cholesterol deposition and macrophage accumulation in the arterial wall (Charo and Taubman, 2004; Zhang et al., 2012; Hilgendorf et al., 2015; Bobryshev et al., 2016). Excessive oxidized LDL (oxLDL) and cholesterol esterification result in the formation of foam cells, which subsequently generate atheromatous plaques (Moore K. J. et al., 2013; Yu et al., 2013; Chistiakov et al., 2016). These plaques can rupture and hemorrhage, which leads to severe conditions including myocardial infarction, peripheral artery disease, stroke, and kidney dysfunction (Swirski and Nahrendorf, 2013). Further, this complex process involves the interaction of pathological mediators (e.g., oxLDL) with arterial wall constituents, such as endothelial cells (Mai et al., 2013; Yin et al., 2015; Li X. et al., 2016; Xi et al., 2016; Li et al., 2017, 2018a; Shao et al., 2020), monocyte-derived macrophages, immune cells (including T cells) (Pastrana et al., 2012; Li et al., 2018b), and vascular smooth muscle cells (Galkina and Ley, 2009). Consequently, atherosclerosis has been defined as a metabolic and immune disease that involves multiple cell types (Paoletti et al., 2006; Galkina and Ley, 2009; Sun et al., 2020; Zhong et al., 2020), as well as the liver (Liu and Czaja, 2013; Fargion et al., 2014; Palmal et al., 2014).

Numerous studies indicate that macrophages are crucial for the development of atherosclerotic lesions because they participate in all stages of plaque formation and progression (Yang et al., 2014; Bories and Leitinger, 2017; Groh et al., 2018). In the early stages, circulating monocytes migrate from the blood stream to the arterial intima. Locally polarized macrophage subsets engulf accumulated oxidized lipids, and become foam cells, which accumulate at the lesion sites and, eventually, cause the failure of plaque resolution. Therefore, monocytes/macrophages are central to lesion formation and the progression of atherosclerosis (Shapiro and Fazio, 2017).

Genome wide-association studies have reported several genes related to cancer biology that are also associated with cardiovascular diseases—suggesting the involvement of epsins in both cancer and atherosclerosis (Holdt and Teupser, 2012). In addition, it has been reported that epsin 1 binds to LDLR to facilitate LDLR internalization through an FxNPxY-independent mechanism in *Caenorhabditis elegans* (Kang et al., 2013). Studies from our laboratory show that epsins are upregulated in lesional macrophages. Using engineered myeloid cell-specific epsin 1 and 2 knock-out mice (LysM-DKO) on an ApoE^–/–^ background and fed a “Western Diet,” these mice display a dramatic reduction in atherosclerotic plaque size and lesion number as well as decreased immune cell infiltration in the aorta and reduced necrotic core formation with an increase in smooth muscle cell number in the aortic root (Brophy et al., 2019). *In vitro* studies demonstrate the absence of epsins inhibited foam cell formation and reduced M1 phenotype macrophages, but increased M2 phenotype macrophages. We also observed a pro-atherogeneic role for myeloid-specific epsins that was resulted from a downregulation of LRP-1. LRP-1 is known to have anti-atherosclerotic and anti-inflammatory functions, which is mediated by the interaction of the epsin UIM domain with LRP-1. With the treatment of oxLDL, the ubiquitination of LRP-1 was markedly increased, resulting in the enhanced interaction between LRP-1 and epsins 1/2. Genetic reduction of LRP-1 in ApoE^–/–^/LysM-DKO-LRP1^f*l/*+^ mice restored atherosclerosis and confirmed the interaction between LRP1 and epsins in atherosclerosis (Brophy et al., 2019). These findings suggest that myeloid-epsin-mediated LRP-1 downregulation plays a vital role in promoting atherogenesis.

In another study, we found that the inducible deletion of epsins 1 and 2 in endothelial cells significantly attenuated atherosclerosis (Dong et al., 2020). Using cultured aortic endothelial cells from double knock-out (DKO) mice treated with atherogenic cholesterol, we discovered that epsins interact with ubiquitinated inositol 1,4,5-trisphosphate receptor type 1 (IP3R1) to promote the degradation of this calcium release channel (Dong et al., 2020). Furthermore, we confirmed that the binding of epsin to IP3R1 in atherogenic conditions occurred through the UIM and N-terminal suppressor domain (SD) of these proteins, respectively. These findings established the role of epsins in endothelial cell dysfunction and the initiation and progression of atherosclerosis.

## Dab2-Deficient Animal Models

Dab2 is a multi-functional adaptor protein and plays roles in many cell functions, including endocytosis (Morris and Cooper, 2001), cell signaling (Drahos et al., 2009; Schutte-Nutgen et al., 2019), lipid uptake (Morris and Cooper, 2001), cholesterol homeostasis (Eden et al., 2007), and cell adhesion (Rosenbauer et al., 2002). Dab2 is also important in embryonic development as deletion of the *Dab2* gene in mice leads to early embryonic lethality prior to gastrulation (Morris et al., 2002b). Interestingly, conditional deletion of Dab2 in embryonic stem cells did not affect the development of embryos, but showed reduced clathrin-coated pits, decreased transport mediated by the lipoprotein receptor in kidney proximal tubule, and increased serum cholesterol levels, which suggest a regulatory role for Dab2 in embryonic development and lipoprotein receptor trafficking (Morris et al., 2002b). In addition, the endocytosis of megalin (also known as low density lipoprotein-related protein 2) is mediated by Dab2 by binding to NPXY motifs on the receptor (Maurer and Cooper, 2005). Studies showing the rescue of embryonic viability also indicates that Dab2-mediated endocytosis is critical for embryonic development (Maurer and Cooper, 2005).

## Dab2 in Inflammation and Cholesterol Metabolism

Recent studies from Norbert Leitinger’s laboratory found that the expression of Dab2 was increased in M2 macrophages and suppressed in M1 macrophages in both mice and humans (Adamson et al., 2016). Deletion of Dab2 results in a pro-inflammatory M1 phenotype, which suggests that Dab2 regulates macrophage phenotypic polarization and inflammatory signaling by inhibiting the NF-κB pathway by binding to TNF receptor associated factor (TRAF) 6 (Adamson et al., 2016). In other studies, analysis of Dab2-deficient bone marrow revealed increased systemic inflammation and cytokine expression, which led to liver injury; however, the effect of Dab2 deletion in atherosclerosis has yet to be determined (Adamson et al., 2018). Moreover, whether the decreased serum lipids as a result of liver injury could counter the elevated inflammation resulted in pro-inflammatory macrophage accumulation in atherogenesis and whether atherosclerotic lesion formation is impacted because of a myeloid deficiency in Dab2 are poorly understood. Consistent with its role in regulating inflammation, Dab2 was also markedly reduced by toll like receptor (TLR) ligands in a TRIF- and MyD88-dependent manner, resulting in a switch in mucosal dendritic cells from a tolerogenic to a pro-inflammatory phenotype (Figliuolo da Paz et al., 2019). Nevertheless, whether Dab2-mediated modulation of aforesaid inflammation by regulating endocytosis of the plasma membrane cargo is unclear.

On the contrary, Dab2 has been shown to potentially regulate LDL receptor (LDLR) endocytosis, and consequently, LDL uptake and cholesterol metabolism as the PTB/PID domain of Dab2 can bind to the NPXY (Asn-Pro-X-Tyr) motif expressed on LDLR. Lipid uptake is mediated by the LDLR through clathrin-dependent endocytosis and the adaptor proteins Arh and Dab2 can specifically interact with the NPXF motif of LDLR and recruit clathrin/AP-2 to facilitate internalization (Tao et al., 2016b). The latter studies suggest that the deletion of both Dab2 and Arh in liver endothelial cells dramatically elevates serum LDL and cholesterol levels, which is different from a single knockout of either Dab2 or Arh. The authors conclude that Arh and Dab2 work together to regulate hepatic cholesterol synthesis through LDLR endocytosis (Tao et al., 2016b).

**FIGURE 4:**
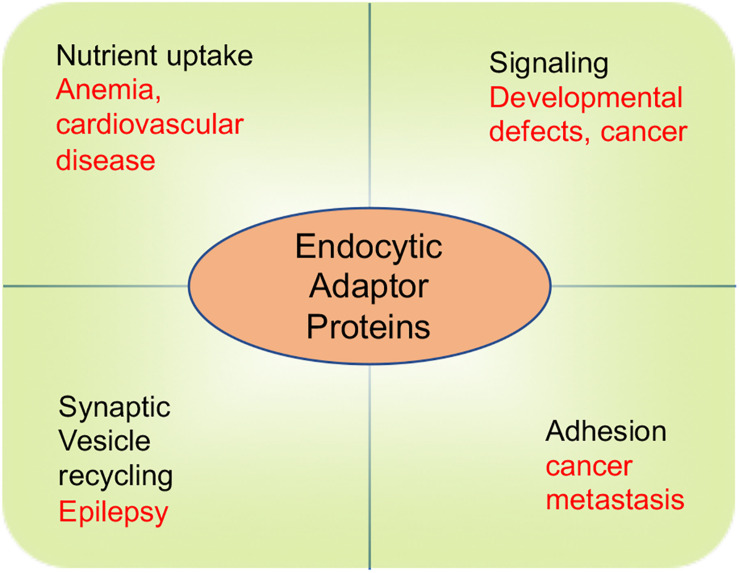
The role of endocytic adaptor proteins in physiological and pathophysiological processes. Physiological processes are depicted by words in black color. Pathological consequences of endocytic defects are depicted by words in red color.

It is notable that despite the multiple physiological roles of endocytic adaptor proteins in governing cell signaling, adhesion, nutrient uptake, and synaptic transmission, malfunction of these proteins cause a suite of endocytic defects that perturb cholesterol homeostasis and inaugurate cardiovascular disease, and produce developmental defects, cancer and neurological disorders (Figure 4). There are numerous molecular mechanisms that remain to be uncovered to determine how these adaptor proteins function. In particular, more work is required to understand how epsins and Dab2 act in a cell context-dependent manner as well as elucidate their interacting partners, post-translational modifications, and their role in the development and progression of diseases.

## Targeting Epsins and Dab2 to Treat Atherosclerosis

Statins remain the most commonly used drugs to treat or prevent atherosclerosis by lowering cholesterol levels in the circulation (Endo et al., 1976; Goldstein and Brown, 2015); although, anti-hypertensive drugs, such as anti-platelet medications, beta blockers, angiotensin-converting enzyme (ACE) inhibitors, and calcium channel blockers are also used to reduce the symptoms of this disease. Unfortunately, due to their potential side effects in multiple organ systems combined with an increased risk for developing diabetes and cancer (Ramkumar et al., 2016), the safety of wide-spread use of statins remains questionable. Consequently, the identification of new therapeutic targets is warranted.

Promising alternatives to reduce atherosclerosis include targeted therapies for the renin-angiotensin system (RAS) and administration of CD47 blocking antibodies to target macrophages (Lu et al., 2008; Kojima et al., 2016). Targeted therapy of PCSK9 in the liver to manage cholesterol levels is another recent alternative (Rader and Daugherty, 2008; Goldstein and Brown, 2015) and Amgen has recently been marketing an antibody to this protein. In addition, anti-inflammatory therapies are being developed to combat atherosclerosis (Rader and Daugherty, 2008; Tabas and Glass, 2013; Li et al., 2019, 2020). In particular, the CANTOS trial clearly suggests that reducing inflammation in patients with prior cardiovascular events using anti-Interleukin-1β antibodies (i.e., Canakinumab) significantly diminishes the risk of recurrent myocardial infarction (Ridker et al., 2017). At the same time, selectively targeting the activated aortic endothelium has proven more difficult despite its obvious potential for treating atherosclerosis.

In our studies, we have shown that endothelial and macrophage epsins are possible therapeutic targets for the treatment of atherosclerosis (Brophy et al., 2019; Dong et al., 2020). Based on the molecular mechanism that we have uncovered, it may be possible to treat this disease by: (1) targeting atheroma-specific epsins using lipid nanoparticle-based delivery of siRNAs to mitigate inflammatory signaling, (2) blocking epsin binding of IP3R1 or LRP1 using UIM-containing peptides in both the endothelium and macrophages, and (3) using an adeno-associated virus gene therapy approach to downregulate epsin expression in lesions.

While several studies show that Dab2 plays an important role in cellular trafficking of LDLR, the relationship between Dab2 and the development of atherosclerosis remains somewhat obscure. A recent study shows that a Dab2 gene variant is associated with increased coronary artery disease risk (Wang et al., 2020). In another study, quercetin attenuated the progression of atherosclerosis by regulating dendritic cell maturation by upregulating Dab2 expression (Lin et al., 2017). Giving the important role of epsins and Dab2 in endocytosis and the regulation of LDLR and LRP trafficking, fully uncovering the regulatory roles of these proteins in different cells and tissues may result in the development of new anti-atherosclerotic therapeutics.

## Perspective

Because of the complexity of atherosclerosis, there have been few effective drugs developed to treat this disease despite decades-long relentless efforts (Weber and Noels, 2011; Libby, 2012; Ridker et al., 2017). As epsins are ubiquitin-binding proteins that play important roles in the vascular system as well as the initiation and progression of atherosclerosis, these endocytic adaptors could represent an important therapeutic target for this disease. Using genetically modified mice, we have demonstrated that the loss of epsins in the aortic endothelium or macrophages inhibits atherosclerosis (Brophy et al., 2019; Dong et al., 2020). Mechanistically, epsins bind IP3R1 in the endothelium and LRP-1 in macrophages via their UIM domain to potentiate atherosclerosis. Targeting epsins in atherosclerotic plaques using liposome nanoparticles containing epsins siRNAs, UIM-containing peptides as competitive inhibitors, or by using a gene therapy approach, may open new avenues to treat atherosclerosis. At the same time, a detailed knowledge of Dab2-receptor interactions in atherosclerosis may identify additional, therapeutically relevant avenues of investigation. In addition, as other endocytic adaptor proteins, such as Numb and Arh, have been implicated in regulating the uptake of cholesterol or cholesterol synthesis (Tao et al., 2016a; Azarnia Tehran et al., 2019). Modulating levels of these endocytic adaptor proteins in cell-restrictive manner would offer equally inspiring opportunity to intervene diseased conditions including dyslipidemia. Thus, developing novel class of drugs that potentially target these endocytic adaptors should offer great promise to circumvent the challenge for the management of dyslipidemia and to reduce cardiovascular diseases.

## Author Contributions

KC, YD, BW, DC, S-LC, JS, and HC wrote the manuscript. KC and HC created the figures. All authors contributed to the article and approved the submitted version.

## Conflict of Interest

The authors declare that the research was conducted in the absence of any commercial or financial relationships that could be construed as a potential conflict of interest.
